# Tackling Acquisition of Language in Kids born Preterm (TALK-Preterm): protocol of a prospective cohort study

**DOI:** 10.21203/rs.3.rs-9077807/v1

**Published:** 2026-09-08

**Authors:** Yuhan Chen, Sara DeMauro, Stephanie Merhar, Devan Polking, Kara Schmidt, Courtney Robinson, Heather Green, Jennifer Vannest, Kristina Ziolkowski, Maria Barnes-Davis

**Affiliations:** Children’s Hospital of Philadelphia; Children’s Hospital of Philadelphia; Cincinnati Children’s Hospital Medical Center; Cincinnati Children’s Hospital Medical Center; Children’s Hospital of Philadelphia; Cincinnati Children’s Hospital Medical Center; Children’s Hospital of Philadelphia; University of Cincinnati College of Medicine; Children’s Hospital of Philadelphia; Cincinnati Children’s Hospital Medical Center

**Keywords:** Language, Development, Neurodevelopment, Prematurity, MEG, MRI, Brain, Connectivity, Children

## Abstract

**Background:**

Children born extremely preterm (EPT) are at significant risk for delays in language development. Language is important for the developing child not only because of its relationship with cognition and academic performance but also because of its impact on quality of life and relationships with peers and caregivers. Late talking, which can be reflective of expressive language difficulties, is particularly common in children born extremely preterm. Preterm infants born at NICHD Neonatal Research Network (NRN) centers have historically been enrolled in the NRN Generic Database (GDB) and followed to 22–26 months corrected age to assess language, motor, and cognitive development with the Bayley Scales of Infant and Toddler Development, 4th edition (Bayley-4) as part of the NRN GDB. For this study, we are recruiting a subset of children from the GDB who were born at < 29 weeks completed gestation and assessed with Bayley-4 at 22–26 months corrected age to undergo additional language assessments, magnetoencephalography (MEG), and MRI at 3 to 4 years corrected age. We are also recruiting a cohort of term-born comparison children (TC) with typical development to undergo the same language and imaging assessments. Our overall objective is to assess the trajectory of – and neural mechanisms underlying – language delay or difficulty in extremely preterm infants, compared to a healthy full-term comparison group.

**Methods:**

The TALK-Preterm study is a prospective follow-up study of EPT infants and term-born peers at two NRN centers that leverages the perinatal and developmental phenotyping data obtained during the first 2 years of life. Children undergo a single in-person study visit at 3–4 years corrected age with multimodal neuroimaging (MEG and MRI) and assessment of language outcomes and parent-completed questionnaires. The term-born comparison group is recruited at 3–4 years and undergoes the same assessment at that time point.

**Discussion:**

The TALK-Preterm study goes beyond standard language assessments by using innovative multimodal neuroimaging to examine the neural mechanisms underlying varied language outcomes in preterm-born children versus to term-born preschool children. By characterizing language trajectories and the neural connectivity that supports language learning, the study will generate rigorous data to advance understanding of variable language outcomes in extremely preterm children and identify brain-based markers of language development that can inform future mechanistic research and promote public health.

## Background

Children born extremely preterm (EPT, defined for this study as less than 29 completed weeks of gestation) are at increased risk for language delay or difficulty versus term-born children, with up to 40% of preterm children experiencing language delays and approximately 20% receiving a diagnosis of specific language impairment^1-5^ or developmental language disorder. Language is important not only because of its association with scholastic performance but also because of its impact on relationships with peers and quality of life. There is still significant uncertainty in predicting long-term language and cognitive outcomes in EPT children with current clinical and research tools^2,6-8^.

The NICHD Neonatal Research Network (NRN) conducts clinical and observational studies in preterm infants in 15 US clinical centers. All infants born at NRN centers at < 27 weeks gestation are eligible for follow-up at 2 years corrected age (CA) to assess language, motor, and cognitive development with the Bayley Scales of Infant and Toddler Development, 4th edition (Bayley-4). This unique cohort provides a rich clinical and phenotyping dataset that is ideal to learn more about the variable trajectories and underlying neural mechanisms of receptive and/or expressive language delay or difficulty associated with prematurity.

Using magnetoencephalography (MEG) and magnetic resonance imaging (MRI), our study team has extensive experience obtaining language neural network measures in awake young children. Whole-brain MEG + MRI provides the ability to directly assess neural activity and functional connectivity between select brain areas with excellent temporal resolution. As an example, our previous findings have reported that increased functional brain connectivity in EPT children, obtained via MEG and MRI, positively relates to language gains from 2 years of age to school age^9^. Other studies from our team have demonstrated that speed of encoding sound in left and right auditory cortices during infancy and toddlerhood is associated with language performance, demonstrating that variability of language skills is in part due to between-child variability in the efficiency of encoding auditory information in the auditory cortex^10,11^.

This article outlines the protocol for the TALK-Preterm study, a prospective follow-up study of extremely preterm infants and their term peers. Built upon our previous research, the present study outlines a comprehensive functional neuroimaging and language assessment protocol to identify novel brain measures sensitive to language outcomes in preterm and term preschool children. Brain measures include auditory encoding as well as the neural networks associated with processing naturalistic speech during stories listening, covert verb generation, and the brain at-rest. We will follow EPT at 3–4 years corrected age to undergo MEG, MRI, and language assessments (Clinical Evaluation of Language Fundamentals, Preschool-3 (CELF-P3) and Peabody Picture Vocabulary Test, 5th edition (PPVT-5)). Parent-completed questionnaires will be administered to obtain socioenvironmental and intervention history data. We will harness the power of neuroimaging (MRI, MEG) to assess the functional connectome supporting language in EPT children at 3–4 years corrected age, comparing those with language difficulties to those without language difficulties. We will also recruit a cohort of term children (TC) without language difficulties to undergo the same protocol at age 3–4 years to compare typically developing TC to children born EPT.

### TALK-Preterm Study Aims

TALK-Preterm will identify neural mechanisms contributing to the heterogeneity in language trajectories in EPT children, improving upon already identified risk factors. The overarching goals are to discover neural measures associated with language difficulties, thus allowing an individualized approach to treatment. TALK-Preterm has three aims (see Fig. 1 for schematic of study aims, hypotheses, and key measures):

Aim 1 will assess the trajectory of language skills in EPT children from 2 years CA (via Bayley-4) to 3–4 years CA (via CELF-P3 and PPVT-5). It is hypothesized that, in EPT children, (a) there will be heterogeneity in the trajectory of language scores, with some children showing improvement and some continuing to experience language difficulties, and (b) the trajectory of language scores will be associated with retrospective clinical measures at neonatal hospitalization, socioenvironmental factors, and intervention history.

Aim 2 will assess the neural basis of language skills in EPT and TC children. We will identify the degree to which the neural measures obtained via MEG (auditory encoding, expressive and receptive language networks, resting-state networks) are associated with language outcomes at 3–4 years CA. It is hypothesized that: (a) EPT children will exhibit delayed auditory M50 latency, increased functional connectivity of language networks, and altered resting-state brain rhythms versus TC children; (b) The degree to which EPT exhibit altered neural measures will be associated with language trajectory from 2 years to 3–4 years CA as well as language scores at 3–4 years CA; (c) MEG markers for auditory, receptive and expressive language processes, and resting-state networks will differentially relate to expressive and receptive language scores as well as general cognitive ability scores in EPT and TC children.

Exploratory Aim 3 will determine if MEG measures improve the model fit for language outcome beyond that predicted by clinical variables. Aim 3 will inform the best prediction model for preschool language outcome.

## Methods

### Study Design

The TALK-Preterm study involves two sites in the US: The Children’s Hospital of Philadelphia (CHOP) and Cincinnati Children’s Hospital Medical Center (CCHMC). Planned recruitment includes 100 extremely preterm (EPT) children born < 29 weeks GA at 3–4 years corrected age (N = 60 at CHOP, N = 40 at CCHMC) and 100 age-matched term-born control (TC) children (N = 60 at CHOP, N = 40 at CCHMC). All EPT children enrolled in the TALK-Preterm study will have had Bayley-4 obtained at 2 years CA. This study leverages the existing EPT cohorts at CHOP and CCHMC, with the same MEG and MRI protocol and the same language and general ability measures collected across the two institutions, with planned enrollment of EPT and TC children using the same inclusion/exclusion criteria.

### Harmonization Approach

The harmonization strategies of the TALK-Preterm study between CHOP and CCHMC include three aspects: (1) Overview of neuroimaging procedures (see Methods section for details of MEG and MRI acquisition protocols and tasks that are optimized for mapping language neural network in preschool children; (2) Phenotyping protocol (including language assessments and parent-report measures of general ability, socio-demographic data, and intervention history); (3) Recruitment strategy for EPT and TC children.

### Study Setting and Procedures

For EPT children, families with children born EPT who are 3–4 years corrected age (CA) will be contacted by phone or email to invite them to participate in TALK-Preterm study. For TC children, recruitment emails will be sent out to families with term-born children at 3–4 years of age who have agreed to be contacted based on the electronic health record (EHR) system at CHOP and CCHMC. Verbal consent and eligibility screening will be conducted over the phone with parents/caregivers in order to collect information needed to plan/prepare for study.

The study will involve a single 4-hour visit to CHOP or CCHMC, with data collection including: language testing, parent-report questionnaires, MEG, and MRI scanning. The order of MEG/MRI imaging sessions and language assessment sessions will be based upon study team’s judgment regarding the best sequence in which to complete the sessions for a particular child.

### Humam Ethics and Consent Declarations

The study is conducted in accordance with the Declaration of Helsinki, and was reviewed and approved by the Children’s Hospital of Philadelphia Institutional Review Board (IRB#23-021618) and the Cincinnati Children’s Hospital Medical Center Institutional Review Board (IRB#2023 – 0594). Informed consent was obtained from all participants.

### Participants and Eligibility Criteria

Inclusion criteria for EPT children are: males or females aged 3–4 years CA, born < 29 weeks GA, with data collected in the CHOP or CCHMC NRN GDB and with a completed Bayley-4 at 2 years CA. As the NRN GDB is a registry that enrolls continuously, all infants who were enrolled in the NRN GDB and are ages 3 to 4 years CA during the study period will be screened for eligibility for the current study.

Inclusion criteria for TC children are males or females aged 3–4 years born full-term (≥ 37 weeks). Additional criteria for TC children recruited at CHOP include birth weight within normal range and no known pregnancy or birth complications.

Exclusion criteria for EPT and TC children include: a diagnosis of autism, no functional hearing, a genetic diagnosis that is expected to be associated with altered language development, primary language other than English, failure to pass standard MRI safety screening (e.g., no VP shunt). For TC children recruited from CHOP, additional exclusion criteria include: having a sibling or parent with an autism or schizophrenia diagnosis, medical diagnoses associated with developmental delay, major congenital heart disease or congenital infection, and uncorrectable sensory (hearing, visual) issues. The EPT and TC children will be matched on age, sex and race at the group level.

### Recruitment

For EPT children, medical history review and screening will be performed by study personnel. The study personnel will reach out to potential participants by phone, email, and text message to invite families and their children to participate in the TALK-Preterm study. Age-matched TC children will be recruited via established methods. TC children will be recruited by the Recruitment Enhancement Core (REC) at CHOP or with the assistance of the Office of Clinical and Translational Research at CCHMC via accessing the EHR at CHOP or CCHMC. REC will contact potential participants directly, on behalf of the investigators using an IRB-approved REC recruitment letter. The investigators will not have access to their names or contact information until interested individuals contact the study team directly. Families may be contacted through REC by email, and the correspondence will contain opt-out language for future communications. At CCHMC, age-matched TC are recruited from community-based research announcements at primary care clinics and CCHMC affiliated sites (such as emergency departments) in the Cincinnati area. Interested families can contact the study team directly using phone or email or scan a QR code to complete pre-screening questions. All families will be screened by study personnel. All families recruited have provided their consent to participate in the study.

### Language and General Ability Assessments

For all children, language outcome measures will be obtained at 3–4 years via direct observation using Clinical Evaluation of Language Fundamentals—Preschool, 3rd Edition (CELF-P3) and Peabody Picture Vocabulary Test, 5th Edition (PPVT-5). The CELF-P3 and PPVT-5 will be administered by trained study personnel (clinical research coordinators and speech-language pathologists). General ability measures will be obtained via the parent-report questionnaire Vineland Adaptive Behavior Scale, 3rd Ed (VABS-3 ^16^). The CELF-P3 is a direct, comprehensive assessment that offers a global view of language skills (i.e., receptive language, expressive language, phonological awareness, and pragmatic skills). The PPVT-5 is used to directly measure receptive vocabulary. Our primary measure of language outcome at 3–4 years will be the Core Language score (CLS) from the CELF-P3. The trajectory of language performance for each EPT child will be assessed using standardized (age-normed) scores on the Bayley-4 (obtained around 2 years CA), CELF-P3 ^17^, and PPVT-5 ^18^ (obtained at 3–4 years CA).

### Prenatal, Perinatal, Socio-demographic, and Intervention History Data

For the EPT children, perinatal, socio-demographic, developmental, and intervention history data are available in the NRN GDB at CHOP and CCHMC and though parent report. For TC, identical perinatal measures will be obtained via CHOP’s and CCHMC’s medical records and parent reports.

### MEG Data Acquisition

MEG data will be collected in a magnetically shielded room using a 275-channel CTF MEG system, the same MEG scanner equipped at both CHOP and CCHMC. MEG approaches were harmonized using the same tasks, stimuli, same hardware for auditory stimuli, acquisition parameters, and MEG acquisition systems. Before the MEG exam, 3 head-position identification fiducial coils will be attached to the child’s head, with the coils activated during the exam so that their position relative to the MEG sensors can be calculated. The acquisition parameters for each MEG task listed below are identical between CHOP and CCHMC, and details of acquisition have been reported in previous publications from the CHOP and CCHMC study teams^9,13,14,19^. Structural MRI (sMRI) will be collected on a Siemens Prisma^™^ 3T MRI System at CHOP or a Philips Ingenia Elition 3T MRI System at CCHMC. Three anatomical landmarks (nasion and right/left preauriculars) will be used for co-registration of MEG data to MRI data; skin-safe markers will be secured at these points during sMRI acquisition to correspond with the MEG coil locations. MEG scan harmonization has been performed by CHOP and CCHMC study investigators, including installing the same MEG tasks on CTF acquisition systems at both sites, completion of timing tests, and human phantom testing at both sites.

### MEG Tasks

The suite of MEG tasks is designed to identify neural markers sensitive to the variable trajectory of language outcome in EPT preschoolers at 3–4 years CA. The primary neural measures of interest include auditory encoding, receptive and expressive language, and resting-state neural networks. All MEG tasks are identical between CHOP and CCHMC MEG sites, with auditory stimuli presented using the same hardware (i.e., speaker). The investigators trained study staff across sites to ensure reliability in administration of MEG tasks.

#### 1. Auditory Encoding:

Auditory evoked responses or AVRs (200 ms pre-stimulus to 500 ms post-stimulus) will be obtained while 500 Hz tones are randomly presented with the inter-stimulus interval ranging from 600 to 2000 ms via a free-field speaker (A total of 300 trials across all conditions). This task was developed by CHOP investigators ^20-22^.

#### 2. Stories Listening:

Five stories will be presented bi-aurally in a female voice, with speech-shaped noise presented between each story. This task was developed and used by investigators at CCHMC ^9,23^.

#### 3. Verb Generation:

Children will be asked to covertly generate a verb or “think of an action word” upon hearing nouns spoken by a female voice (verb generation condition) or to quietly attend to speech-shaped noise stimuli without responding. Prior to MEG recording, participants will be trained on an overt version of the task to assess understanding and promote compliance during acquisition. This task was developed and used by investigators at CCHMC ^13,24^.

#### 4. Resting-State (RS) Dark Room:

Given that young children may not be able to reliably open and close their eyes on demand using a traditional eyes-closed MEG resting-state paradigm, we use an alternate RS paradigm^25,26^. During a 5-minute recording, the children will be awake while the MEG room is kept completely dark except for brief 10 second periods when a silent Inscape movie ^27^ is displayed on a screen. This task was developed by CHOP investigators ^14,15^.

### MEG Data Analyses

#### 1. Time domain auditory evoked-response analyses:

Left and right auditory cortical activity will be obtained to examine auditory encoding processes. A MEG analysis pipeline for infants and young children, developed by CHOP investigators^10,11^, provides evoked source waveforms for a given cortical region. MEG data will be preprocessed and analyzed using Brainstorm^28^. Locations of the HPI coils will be co-registered to the individual’s structural MRI. Artifact-free auditory evoked response fields will be obtained by averaging epochs 200 ms pre-stimulus to 500 ms post-stimulus at each sensor. Whole-brain maps of auditory response activity will be computed using distributed source modeling (e.g., Minimum Norm Estimates (MNE)^29^) and mapped into a cortical atlas as a function of time. A left and right auditory cortex region of interest (ROI) will be identified based on the location of the auditory M50 response^30^, and source timecourses will be computed from the left and right auditory ROI and the M50 peak latency and amplitude identified from each source waveform using in-house software (see details in Chen et al. ^22,31-33^).

#### 2. Functional Connectivity Analyses:

Nodes for functional connectivity analysis will be obtained from age-appropriate canonical language maps and from participants at CCHMC who have completed the same language tasks during fMRI to increase rigor and reproducibility^9,12,13^. MEG data will be analyzed using FieldTrip^34^ routines in MATLAB. Headmodels will be constructed from each individual’s 3D T1 images when available and an age-appropriate template when not. Using a linearly constrained minimum-variance (LCMV) beamformer, we will estimate time series of activity at each node, computing pairwise functional connectivity using debiased weighted phase-lag index (wPLI)^35^. wPLI is a measure of phase difference distribution across trials. Consistent trial-to-trial phase differences are reflected in greater wPLI values, indicating nontrivial functional connectivity between a pair of nodes^35^. We will identify frequencies of interest in the connectivity spectrum and evaluate group differences^36^. Supra-threshold connections will be clustered in topological space and subjected to permutation testing. Graph theoretical metrics, such as network strength, will be calculated from MEG data at 3–4 years and related to language scores at 3–4 years and (for the EPT group) to changes in language scores from 2 to 3–4 years.

#### 3. Resting-State SpecParam analyses:

Resting-state (RS) MEG data will be preprocessed and analyzed using Brainstorm^28^. Source estimation using MNE^29^ is computed for the continuous recording of RS data bandpassed 0.5 to 55 Hz. Power spectrum density (PSD) at the source level (across all vertices of the cortical surface) will be computed for artifact-free RS data in 4 sec epochs (with Hanning window) with 50% epoch overlap. SpecParam^37^ algorithm will be used to compute RS periodic (peak alpha frequency and amplitude) and aperiodic (exponent and offset) measures across different brain regions. For details please see Green et al., (2025)^15^ and Edgar et al. (2023)^14^.

### sMRI Data Acquisition and Analyses

MRI data will be acquired when the child is awake and without sedation. Prior to the MRI, the child will be asked to choose their choice of movie to watch during the MRI scan. During the MRI scan, ear protection will be provided to the child. Each child will undergo 12 min of structural imaging: a T1-weighted, magnetization-prepared rapid gradient-echo (MP-RAGE) and a T2-weighted image, both collected with 0.8x0.8x0.8 mm^3^ isotropic resolution. The T1-weighted data will be processed to extract the brain and head surface (needed for source modeling). Given that the sMRI data will be primarily used for MEG source reconstruction as well as to identify and control for clinical findings in EPT children, MRI scan harmonization is not needed. However, we have adapted the HBCD T1-weighted and T2-weighted protocol for the TALK-Preterm study across both sites, with the harmonization across scanner platforms performed under the HBCD Study protocol^38^.

### Statistical Analyses Plan

Aim 1 statistical analyses will be performed only in the EPT cohort. For Aim 2 and exploratory Aim 3, we will combine the EPT and TC cohorts from CHOP and CCHMC (N = 60/per group) and CCHMC (N = 40/group). Assuming 15% data loss (missing or non-evaluable data due to children unable to tolerate scan procedures), we anticipate evaluable MEG and language outcome data from 85 EPT children and 85 TC children.

#### 1. Aim 1:

Mixed-effect models will be used to determine the degree to which clinical and developmental measures (i.e., Bayley-4 language scores) obtained during neonatal hospitalization and at 2 years CA correlate with language outcome at 3–4 years CA and/or the language trajectory from 2 to 3–4 years CA. Language outcome measures at 3–4 years CA in EPT children include composite/core, receptive, and expressive language scores. Separate mixed-effect models will be used for each language domain score. For example, to determine if Bayley-4 language score accounts for significant variance in CELF-P3 Core Language scores after accounting for clinical measures (e.g., birth weight and GA), a mixed-effect model will be run with Core Language standard score as a dependent variable, birth weight, GA, and their interactions entered as fixed-effect variables, and subjects as a random-effect variable.

#### 2. Aim 2:

Group differences (EPT versus TC) for each MEG measure (Fig. 1) will be performed separately. A group x sex x hemisphere ANCOVA will examine group differences in the MEG measures, with age entered as a covariate. Hemispheric specialization of auditory and language neural networks is of interest, with the group x hemisphere interaction assessing EPT and TC difference in hemisphere lateralization. Of note, prior to ANCOVA, for functional connectivity measures, we will identify frequencies of interest in the connectivity spectrum and evaluate group differences using Network Based Statistics (NBS). Supra-threshold connections will be clustered in topological space and subjected to permutation testing. To evaluate if each MEG measure is associated with language outcome at 3–4 years CA in EPT and TC children, mixed-effect models will be used to determine the degree to which each MEG measure accounts for significant variance in language scores (see Fig. 1). As an example, a mixed-effect model will be run with CELF-P3 Core Language score entered as a dependent variable, M50 latency, group, hemisphere, sex, and their interactions entered as fixed-effect variables, and subjects entered as a random-effect variable. For functional connectivity measures, graph theoretical metrics, such as network strength, will be calculated from the MEG data and related to language scores and (for the EPT group) to changes in language scores from 2 to 3–4 yrs.^12^

#### 3. Exploratory Aim 3:

To determine if the MEG measures account for variance in language outcome beyond that accounted for by other risk variables obtained, a baseline mixed-effects model will be created with language outcome (e.g., Core Language scores) as the dependent variable, with risk factors as fixed-effect variables (e.g., prenatal, perinatal, parental, and sociodemographic variables), and with subjects as a random-effect variable. Each MEG measure will then be added to the baseline model to determine whether MEG measures improve the model fit via comparing adjusted r^2^ values to the baseline model. For each MEG variable, the model with the largest adjusted r^2^ will be selected. Analyses are expected to show that the brain measures predict variance in language outcome beyond that accounted for by the available clinical data, thus demonstrating that the neural measures provide unique information. Analyses can also be run in reverse, entering the MEG measures first and the risk factors second to determine the unique variance for the risk factors.

### Power Analyses

An overarching study goal is to identify associations between neural measures and language ability. The neural basis of language in prematurity is poorly understood, as is the relationship between brain-based markers of language function and environmental factors that could be targeted to improve later language outcomes. For Aim 1, we anticipate language scores at 3–4 years (and the change in language scores from 2 years CA to 3–4 years) will relate to social and clinical factors for the EPT group. We will use a mixed model to relate language scores to social and clinical variables. For Aim 2 we anticipate our suite of neural markers will differentially relate to language for EPT and TC. For EPT, we expect auditory encoding and functional hyperconnectivity (versus TC) in a more bilateral, temporal network to be positively related to language scores and language trajectories from 2 to 3–4 years. This is informed by our preliminary studies, which we used to estimate power to detect brain-language associations of the size we have previously observed. For example, in Chen et al.^22^ the CHOP investigators observed an association of *r* = −0.63 (N = 114 ^22^) between left auditory evoked response latency and language ability and associations of *r* = 0.58 to 0.73 between left M50 latency and language scores. In Barnes-Davis et al. (2021) the CCHMC team demonstrated a correlation of *r* = 0.32 (N = 31 EPT)^13^ between gamma band connectivity during verb generation and expressive language scores in EPT children at 4–6 years of age Furthermore, when the CCHMC investigators related the change in standardized language scores from 2 years CA to 4–6 years to network strength during stories listening, they found a correlation of *r* = 0.62 (N = 19 EPT)^9^. Thus, the proposed sample of children provides more than sufficient power to identify similarly sized associations with at least 0.80 power. In particular, when alpha = 0.05, power is 0.80 to detect (1) correlations ≥ 0.33 if we were to run analyses on only ~ 50 EPT and ~ 50 TC children, and correlations as low as 0.26 if 85 TC and 85 EPT children are evaluable combined across CHOP and CCHMC.

## Discussion

The TALK-preterm study is currently recruiting preterm and term children at two clinical sites and will provide important information on the impact of prematurity on the trajectory of language development in the toddler and preschool years, neural measures that underlie language development, and the relationship of language development to socioeconomic factors and therapies received. There is a relative paucity of neuroimaging studies investigating brain-based mechanisms of language delay or disorder in preschool and school-age children born preterm, a dynamic period for language acquisition and literacy development. Research that has been performed in these age groups has largely relied on structural and diffusion MRI obtained at term-born age^39-42^. For this study, we have chosen to focus on MEG as the primary neural marker of language development because it offers millisecond-level temporal resolution, allowing precise tracking of the rapid neural dynamics that underlie speech and language processing. MEG is also noninvasive and relatively well-tolerated by children, making it well-suited for developmental studies. In addition, its spatial resolution for cortical activity will enable us to link language skills to specific neural networks^9,11-15,33^. The auditory encoding task selected for this study has been used in MEG studies across the lifespan by our study team. In particular, the left and right auditory M50 latency as well as the lateralization index of M50 latency were reported to be associated with the language skills in typically developing infants and toddlers^10^. Left M50 latency was also reported to be associated with language outcome in preschool children at risk for intellectual disability^43^, and as a potential biomarker for children with neurodevelopmental disorders that are associated with language delay (e.g., autism)^30^. The receptive and expressive language tasks selected for this study have been used in MEG and fMRI studies of term and preterm children by our study teams (as well as other groups) and are among the most widely used and recommended tasks for pre-neurosurgical mapping of eloquent cortex in adults and children^9,13,44-48^. The resting-state task selected for this study has been developed by our study team, with resting-state periodic and aperiodic measures shown to be associated with general ability and processing speed, an indication of general cognitive ability in pediatric populations from infancy to school ages^14,15,49^. The rationale for these tasks is that they will index distinct language functions. Furthermore, they will increase our knowledge of the neural basis for language development and delay. Finally, they will establish feasibility of MEG in a younger pediatric population than we have previously tested, during critical ages when language emerges rapidly.

Few studies have evaluated language trajectories over time in preterm infants and related them to other risk factors. We will include preterm infants with scores on the Bayley-4 receptive and expressive language subscales and then collect the CELF-P3 and PPVT-5 at 3–4 years CA, providing language development measures collected at least one year apart during a period of rapid language growth. We hypothesize that there will be heterogeneity in the trajectory of language scores in EPT with some children showing improvement and some continuing to experience language difficulties, and that the trajectory of language scores in EPT will be related to socioenvironmental factors and therapies received.

We chose to use the CELF-P3 and PPVT-5 as our measures of language development in this study. Both sites in this study have experience with using these measures in older preschoolers and thus have staff trained on these assessments. The CELF-P3 provides a comprehensive assessment of both receptive and expressive language abilities across multiple domains relevant to early communication. It has strong psychometric properties and is widely used in clinical and research settings, supporting reliable comparisons across studies and developmental trajectories. The PPVT-5 provides a well-validated, age-appropriate assessment of receptive vocabulary, a core component of early language development. It places minimal demands on speech production, attention, and motor skills, making it especially suitable for young children and for populations with developmental or medical risk. In addition, the PPVT-5 has strong normative data and reliability, allowing researchers to compare individual performance to age-matched peers and to track language outcomes across studies. The PPVT has also been used in longitudinal studies of language in preterm children by other investigators.^7,50,51^ Using both measures in this multicenter study will allow us to assess expressive and receptive language ability in EPT and TC children.

Leveraging the unique resource and facilities at CHOP and CCHMC, this is the first study utilizing harmonized MEG, MRI, and language assessment protocols to study neural circuit contributing to variable language outcome in extremely preterm preschool children. Although differences in hardware (e.g. CHOP has a Siemens 3T MRI scanner and CCHMC has a Philips 3T MRI scanner) necessitated differences in MRI acquisition parameters, protocols were harmonized carefully using the approach of the HBCD study^38^. Despite careful harmonization, differences may remain which may introduce site-related variability in the data and require explicit modeling or sensitivity analyses during the data analysis phase. In this protocol paper, we have documented both the harmonized elements and residual protocol differences to strengthen reproducibility.

## Supplementary Material

This is a list of supplementary files associated with this preprint. Click to download.

• STROBEchecklistv4combinedPlosMedicineNRNTALKProtocol.pdf

## Figures and Tables

**Figure 1:**
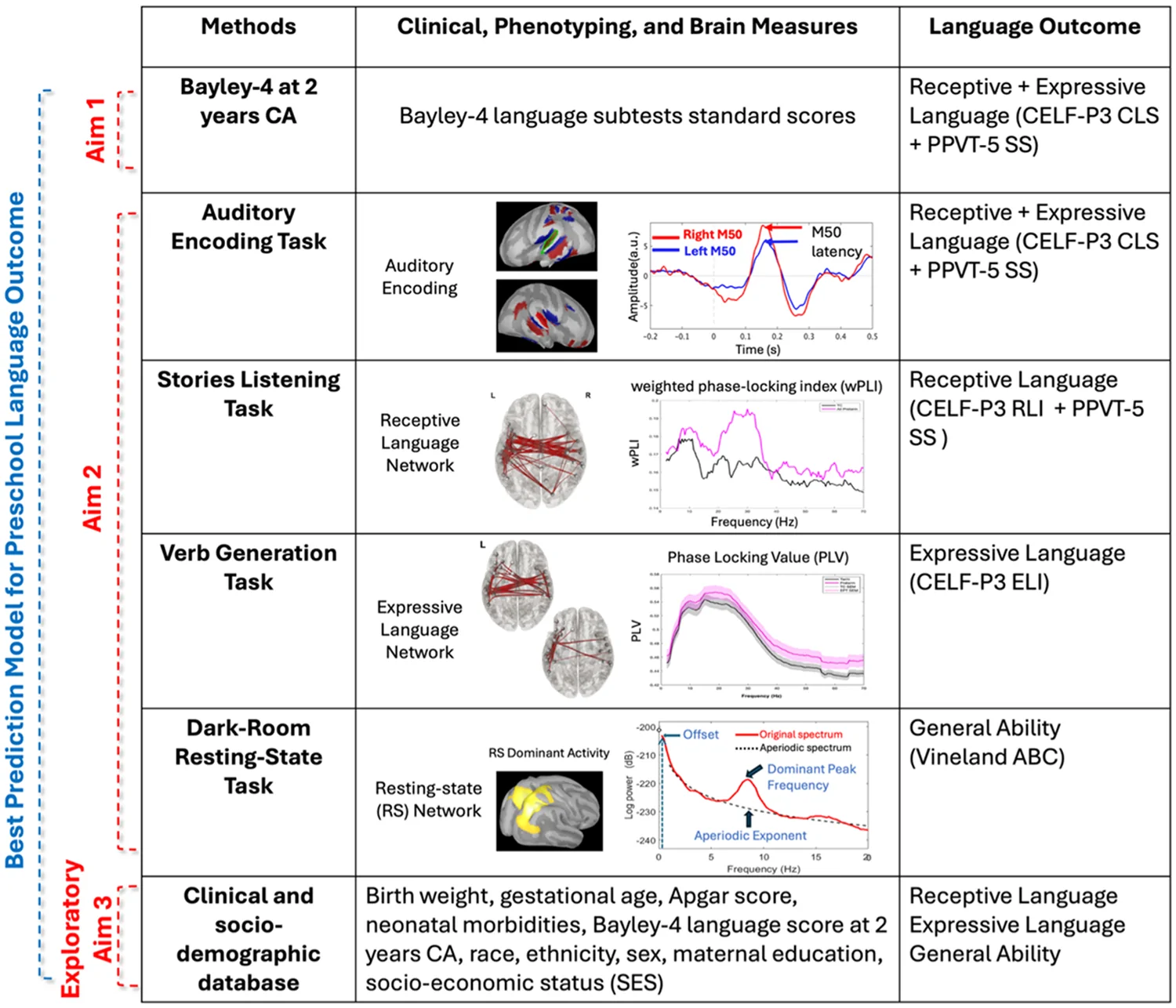
Schematic of TALK-Preterm study hypotheses, study aims, and key language and neural measures of interest. Language and General Ability outcome measures are obtained from: (1) CELF-P3: Core Language Score (CLS), Receptive Language Index (RLI), Expressive Language Index (ELI), (2) PPVT-5 standard score (SS), and (3) Vineland Adaptive Behavior Scales 3^rd^ edition - Adaptive Behavior Composite score (Vineland ABC). Please see details of brain measures in study team’s publications: Auditory Encoding is detailed in Chen et al., (2023)^10,11^; Stories Listening is detailed in Barnes-Davis et al. (2018)^12^; Verb Generation is detailed in Barnes-Davis et al. (2021)^13^; Resting-State is in Edgar et al. (2023)^14^ and Green et al. (2025)^15^.
